# Relationship between Depression and Physical Activity Frequency in Spanish People with Low, Medium, and High Pain Levels

**DOI:** 10.3390/jpm14080855

**Published:** 2024-08-12

**Authors:** Ángel Denche-Zamorano, Diana Salas-Gómez, Jose A. Parraca, Pablo Tomas-Carus, José Carmelo Adsuar, Sabina Barrios-Fernandez

**Affiliations:** 1 Promoting a Healthy Society Research Group (PHeSO), Faculty of Sport Sciences, University of Extremadura, 10003 Cáceres, Spain; denchezamorano@unex.es; 2 Departamento de Desporto e Saúde, Escola de Saúde e Desenvolvimento Humano, Universidade de Évora, 7004-516 Evora, Portugal; jparraca@uevora.pt (J.A.P.); ptc@uevora.pt (P.T.-C.); 3 Escuelas Universitarias Gimbernat (EUG), Physiotherapy School Cantabria, University of Cantabria, 39300 Torrelavega, Spain; 4 Comprehensive Health Research Centre (CHRC), University of Evora, 7004-516 Evora, Portugal; 5 Interdisciplinary Centre for the Study of Human Performance (CIPER), Faculty of Human Kinetics, University of Lisbon, 1649-004 Lisbon, Portugal; 6 Social Impact and Innovation in Health (InHEALTH), Faculty of Sports Sciences, University of Extremadura, 10003 Cáceres, Spain; sabinabarrios@unex.es

**Keywords:** mental health, exercise, psychological distress, quality of life, depression

## Abstract

Depression is a mental disorder that causes great discomfort, is associated with unhealthy lifestyle habits, and affects the quality of life. People with pain show high depressive symptoms and a prevalence of physical inactivity. This study aimed to analyse the associations between depression (self-reported depression, depression status, depressive symptoms, and depression types) and physical activity frequency (PAF) in leisure time in middle-aged and older people with different pain levels (low, medium, and severe) living in Spain. A cross-sectional study based on the European Health Survey data from Spain (2014–2020) was carried out, with a final sample of 13,686 people with pain. Associations between depression-related variables and PAF were studied at the three levels of pain, comparing the prevalence of depression as a function of PAF. Regression models were performed to assess adjusted risk factors for depression (self-reported depression and depression status). It was found that PAF was related to depression at all three pain levels. Inactive people at each pain level had higher depression prevalence (self-reported depression, depression status, depression symptoms, and depressive types). Physical inactivity emerged as a risk factor for depression, both for self-reported depression and for depression status. Thus, increased PAF could help prevent or reduce depression and depressive symptoms in people with pain.

## 1. Introduction

Depression is a mood disorder that causes a persistent feeling of sadness and loss of interest, associated with a reduced quality of life, comorbidities, and higher mortality [1]. This disorder is associated with an incremental economic burden on health care systems [2,3], but it is also related to indirect costs due to productivity loss and unemployment, as well as due to co-morbidities, with chronic illnesses causing a great economic burden [4]. The World Health Organization (WHO) estimates that 3.8% of the population experience depression, including 5% of adults (4% among men and 6% among women) and 5.7% of adults older than 60 years. It is more common among women than among men [5]. On the other hand, pain is defined as “an unpleasant sensory and emotional experience associated with, or resembling actual or potential tissue damage”, and it is an experience influenced by life experiences and biological, psychological, and social factors [6]. The sensation of pain is divided into four main types: acute pain, nociceptive pain, chronic pain, and neuropathic pain [7]. Pain may have adverse effects on people who experience it at the psychological and social levels, impacting their well-being, although, in principle, its function should be adaptive [6].

Currently, the recommended interventions include psychotherapy and antidepressant medication or a combination of both [8]. Nevertheless, exercise has proven to be an effective adjunct treatment for people with depression, including benefits on physical and mental health [9]. Exercise can cause changes in the levels of the neurotransmitters (serotonin, dopamine, and norepinephrine) involved in mood regulation; it can also trigger the release of endorphins, natural opioids produced by the body that can reduce pain and promote feelings of well-being; moreover, exercise can provide opportunities for social interaction and social support [10]. In addition to producing therapeutic benefits, it also helps in preventing or delaying the onset of mental disorders [11]. Thus, clinical practice guidelines from the USA, UK, and Australia issued by organisations such as the WHO [12], the National Institute for Health and Care Excellence (NICE) [13], or the American Psychiatric Association (APA) [14], among others, recommend physical activity as part of depression management [8]. Although the exact relationship between exercise and pain is still unknown, acute exercise produces a hypoalgesia period in which sensitivity to painful stimuli decreases [15]. Therefore, exercise has a preventive role in the development of chronic pain and pain associated with different diseases [15]. Moreover, the results improve when implemented as part of a multimodal treatment. In the case of low back pain, recent systematic reviews conclude that there is a marked heterogeneity in the recommendations regarding physical activity and targeted exercise [16,17]. In the case of chronic cervical pain, no effects were found; in hip and knee osteoarthritis, short- and medium-term benefits were found, and benefits were found in rheumatoid arthritis [15].

On the other hand, in recent years, there has been a global health crisis with many consequences. According to the World Health Organization (WHO), in the first year of the COVID-19 pandemic, the global prevalence of anxiety and depression increased by 25%; however, these data may continue to increase, and more research is needed [18]. There is therefore a global need for all countries to pay more attention to mental health and to do a better job of supporting and preventing mental health in their populations.

However, to the best of the authors’ knowledge, as of 2024, only one study has estimated the probability of chronic pain patients experiencing depression, and that study was conducted on participants in the United Kingdom [19]. No studies have been carried out in a representative Spanish sample of middle-aged and older people to assess the relationship between depression, pain, and physical activity. Therefore, the main objective of this study was to verify the dependence relationships between physical activity frequency and self-reported depression, depression status, depressive symptoms and types of depression, measured using the Patient Health Questionnaire-8, in middle-aged and older adults with low, medium and high pain. The secondary objective was to compare the proportions of variables related to depression as a function of physical activity frequency in each pain group. Also as a secondary objective, we analysed the risk factors for depression adjusted for sociodemographic variables, life habits like physical activity, and social support.

## 2. Materials and Methods

### 2.1. Study Design

A secondary cross-sectional study was conducted based on primary published data from the 2014 and 2020 European Health Survey (EHIS) carried out in Spain. According to Regulation 2016/679 of the European Parliament and of the European Council of 27 April 2016 on the protection of individuals concerning the processing of personal data and on the free movement of personal data and derogating from Directive 95/46/EC, these data are anonymous, public, and considered non-confidential. Therefore, it was not necessary to apply data protection principles, and the approval of an accredited ethics committee was not necessary. The STROBE recommendations were followed in this study.

### 2.2. Instruments

The European Health Survey (EHIS) in Spain aimed to measure the health status and health determinants (lifestyle-related, such as sedentary lifestyles) of European Union citizens, as well as their use of and limitations to access of health care services, in a harmonised way and with a high degree of comparability between Member States (MSs). Coordinated by the European Statistical Office (Eurostat), it was carried out by those responsible for each MS; in the case of Spain, by the National Statistics Institute (INE) in collaboration with the Spanish Ministry of Health (SMH). The questionnaire consisted of four modules: health status, health determinants, health care, and background (sociodemographic) variables. In Spain, interviewers trained and accredited by the INE administered the questionnaire through in-person interviews. Data from the EHIS 2014 (between January 2014 and January 2015) and the EHIS 2020 (between July 2019 and July 2020) were used. Previously, participants had been selected using a stratified random sampling system; they were informed of their selection, the nature of the survey, and the anonymous treatment of the data, and consent was obtained from those who agreed to participate. The methodologies of both surveys can be found here: EHIS 2014 https://www.sanidad.gob.es/en/estadEstudios/estadisticas/EncuestaEuropea/Enc_Eur_Salud_en_Esp_2014.htm (accessed on 1 July 2024) (https://www.sanidad.gob.es/en/estadEstudios/estadisticas/EncuestaEuropea/Enc_Eur_Salud_en_Esp_2014.htm, (accessed on 1 July 2024)) and EHIS 2020 (https://www.sanidad.gob.es/en/estadEstudios/estadisticas/EncuestaEuropea/Enc_Eur_Salud_en_Esp_2020.htm, (accessed on 1 July 2024)).

### 2.3. Sample

Data from and responses to the questionnaires can be freely downloaded from the website of the INE (https://www.sanidad.gob.es/en/estadisticas/microdatos.do, (accessed on 1 July 2024)). The EHIS 2014 and 2020 had a final sample of 22,842 and 22,072 participants, respectively. All of them were individuals aged 15 and over living in family dwellings in Spain.

**Figure 1:**
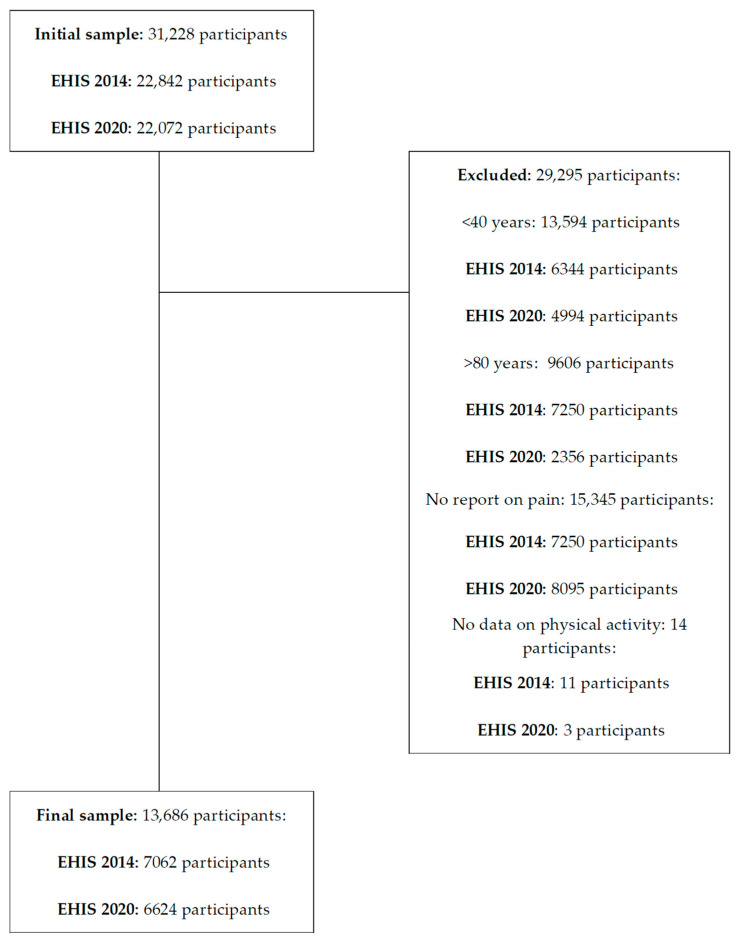
Flow chart including the sample eligibility criteria.

Once downloaded, the following inclusion criteria were applied to reach the final sample for the present study: (1) being a middle-aged adult and older (between 40 and 80 years old) with pain; (2) reporting data on variables related to physical activity frequency. After applying these criteria, in the EHIS 2014, 6344 persons aged <40 years and 7250 aged >80 years were excluded; a further 7250 were excluded due to not having pain or not reporting data on pain and 11 due to not reporting data on physical activity frequency. In the EHIS 2020, we excluded 4994 persons aged <40 years and 2356 aged >80 years; a further 8095 were excluded due to not having pain or not reporting data on pain and 3 due to not reporting data on physical activity frequency. A final sample of 13686 participants was obtained: 7062 and 6624 in the EHIS 2014 and 2020, respectively. Figure 1 shows a flow chart of the sample selection process.

### 2.4. Variables Extracted from the European Health Survey in Spain

#### 2.4.1. Outcome Variables

Variables related to depression

*Self-reported depression (S-RD*): This variable was obtained from responses to item 25a.20. The question was “Do you have, or have you ever had Depression?”, with possible answers as follows:

1. Yes.
2. No.
3. Don’t Know or Don’t Answer (DK/DA).

*PHQ-8 symptoms:* This variable was derived from the EHIS variable “Depressive Severity”. To assess the prevalence of depression, the EHIS relied on the Patient Health Questionnaire (PHQ-8). The PHQ-8 is a valid 8-item instrument used to diagnose and assess the severity of depressive disorders [20]. Participants were asked how often they have experienced the following series of situations in the last two weeks:

A. Little interest or joy in doing things.
B. Feeling down, depressed or hopeless.
C. Trouble falling asleep, staying asleep or sleeping too much.
D. Feeling tired or having little joy.
E. Poor appetite or eating too much.
F. Feeling bad about yourself, feeling that you are a failure or that you have let yourself or your family down.
G. Trouble concentrating on something, such as reading the newspaper or watching television.
H. Moving or talking so slowly that others may have noticed. Or the opposite: being so restless or agitated that you have been moving around more than usual.

For these statements, the response options were “Never”, “Several days”, “More than half of the days”, or “Almost every day”. These answers were given a value from 0 (Never) to 3 (Almost every day) points, and values for the 8 items were added together to give a score between 0 and 24 points. According to the total scores, the following symptom severities were obtained: None (less than 5 points), Mild (5 to 9 points), Moderate (10 to 14 points), Moderately severe (15 to 19 points), and Severe (more than 19 points). In this study, participants were grouped into the following categories according to their symptoms:

1. None (less than 5 points).
2. Mild (5 to 9 points).
3. Moderate to Severe (more than 10 points).

*PHQ-8 depression types:* This variable was obtained from the EHIS variable “Depressive Conditions”, and it was constructed from the answers given to the PHQ-8, grouping participants into two groups:

1. Major depression: if participants had five or more symptoms, including anhedonia and feeling depressed, with a frequency of more than half of the days.
2. Other depression: if participants had between two and four symptoms, including anhedonia or feeling depressed, with a frequency of more than half of the days, or none (less than two symptoms).

*PHQ-8 depression status:* This was a dichotomous variable derived from the previous variable. It was created by grouping participants according to whether or not they had any type of depression:

1. Yes: participants with major or other depression.
2. No: participants without major and other depression.

#### 2.4.2. Independent, Predictor, and Covariate Variables

*Age:* in years, drawn from the “AGEa” EHIS variable.

*Sex:* taken from the “SEXOa” variable in the survey, with two possible responses (men or women).

*Body Mass Index (BMI) group*: drawn from the variable “BMIa”. Participants were grouped according to their BMI (weight in kg/height^2^ in metres). The following categories were established:

1. Underweight (BMI < 18.5).
2. Normal (BMI ≥ 18.5 and <25).
3. Overweight (BMI ≥ 25 and <30).
4. Obesity (BMI ≥ 30).

*Physical activity frequency (PAF) in leisure time:* drawn from item Q.112, “Which of these possibilities best describes the frequency with which you do some physical activity in your free time?”, with four response options:

1. I do not exercise. I spend my free time almost sedentary (“Inactive”).
2. I do some occasional physical activity or sport (“Occasionally”).
3. I do physical activity several times a month (“Frequently”).
4. I do sports or physical training several times a week (“Very Frequently”).

*Pain level:* drawn from item P45, “During the last 4 weeks, what level of pain have you experienced?”, with six possible levels: “None”, “Very mild”, “Mild”, “Moderate”, “Severe”, and “Extreme”. The responses were grouped as follows:

1. None: those who responded “None”.
2. Low: those who responded, “Very Mild” or “Mild”.
3. Medium: those who responded “Moderate”.
4. Hight: those who responded “Severe” or “Very severe”.

*Social class:* based on item CLASE_PR, which grouped the participants into 6 categories according to the 2011 National Classification of Occupations (CNO 2011): I, II, III, IV, V, and VI) [21]. More detailed descriptions can be found in Table S1.

*Smoking status:* drawn from item Q.121, “Can you tell me if you smoke?”, with 4 levels:

1. Yes, I smoke daily (“Smokers”).
2. I do not currently smoke, but I have smoked before (“ExSmokers”).
3. I do smoke, but not daily (“Occasionally smokers”).
4. I do not smoke and have never smoked regularly (“Non-Smokers”).

*Social support:* this variable was assessed in the EHIS using the Oslo Social Support Scale (OSSS-3) [22], consisting of three questions (items 130, 131, and 132). Item 130, “If you had a serious personal problem of any kind, how many people could you count on?”, had 4 possible answers: none, 1 to 2 persons, 3 to 5 persons, and more than 5 persons. Item 131, “To what extent are other people interested in what is happening to you?”, had 5 possible answers: a lot, some, not very much, not very little, little, and not at all. And item 132, “How easy would it be for you to get help from neighbours in case of need?”, had 5 answers as well: very easy, easy, possible, difficult and very difficult. The result of the scale was the sum of the scores of all questions (*OSSS-3 sum*). The range was between 3 and 14 points.

In addition, to form the *Oslo level* variable, the results were ranked as follows:

1. Between 3 and 8: poor social support (“Poor”).
2. Between 9 and 11: moderate social support (“Moderate”).
3. Between 12 and 14: high social support (“Hight”).

Participants who did not submit data on any of these variables were excluded from the analyses including these data variables, although they were included in the rest of the analyses.

### 2.5. Statistical Analysis

The normality assumption of the continuous variables was checked using the Kolmogorov–Smirnov test. Descriptive analysis of continuous and categorical variables was performed using medians (Ms) and interquartile ranges (IQRs) and frequencies and proportions, respectively. These data were presented for the whole sample and stratified by sex. The Mann–Whitney U-test was used to check differences in continuous variables (age and OSSS-3 sum) between men and women. The chi-squared test was used to analyse dependence relationships between sex and all categorical variables and between PAF in leisure time and depression-related variables. This second analysis was performed at each pain level (low, medium, and high) and stratified by sex to present associations between PAF, depression variables at each pain level, and sex. In both cases, the post hoc pairwise z-test for independent proportions was performed to check differences in proportions. The phi and Cramer’s V coefficients were calculated to interpret the strength of these associations.

Finally, a multiple binary logistic regression was performed to avoid or reduce confounding biases when analysing the odds of depression status or self-reported depression, with the following independent variables: PAF as the main predictor and adjusted for the other covariates, i.e., sex, age, social support, smoking, social class, BMI, and pain level. Depression status and self-reported depression were included as dependent variables. The model was run for each dependent variable. Their adjusted probability risks and 95% confidence intervals were calculated. For multiple logistic binary regressions, the assumptions of the absence of influential factors, independence, and non-collinearity were tested. IBM SPSS Statistics for Windows Version 27.0 (IBM Corp, Armonk, NY, USA) software was used and a value of *p* < 0.05 was considered significant.

## 3. Results

### 3.1. Sample Characterisation

The main sociodemographic variables characterising the sample are presented in Table 1. The normality test results showed that the continuous variables in this study did not follow a normal distribution (*p* < 0.001).

The median age of the participants was found to be 60 years and was slightly higher in women than in men (60 vs. 59, *p* < 0.001). Participants had strong social support, with a median of 12 points in both sexes (*p* = 0.001). Dependence relationships were found between sex and all categorical variables in this study: social support, pain level, S-RD, PHQ-8 DS, depression symptoms, depression types, PAF, BMI, smoking status, and social class (*p* < 0.05; 0.022 ≤ V ≤ 0.293).

Men had a higher prevalence of low pain levels (56.1% vs. 44.6%, *p* < 0.001), while women had a higher prevalence of medium (34.6% vs. 30.2%, *p* < 0.001) and high (20.9% vs. 13.7%, *p* < 0.001) pain levels.

Women had higher proportions of S-RD (24.0% vs. 13.0%, *p* < 0.001) and PHQ-8 DS (15.6% vs. 9.5%, *p* < 0.001), depressive symptoms (mild—18. 8% vs. 13.2%, *p* < 0.001; moderate to severe—12.6% vs. 7.2%, *p* < 0.001), and depression types (major—7.6% vs. 4.4%, *p* < 0.001; other depression—8.1% vs. 5.2%, *p* < 0.001). In addition, women had a higher prevalence of physical inactivity in leisure time (42.4% vs. 37.9%, *p* < 0.001), while men had higher proportions of PAF (frequently (8.9% vs. 7.2%, *p* < 0.001) and very frequently (10.3% vs. 8.6%, *p* = 0.001)).

### 3.2. Depression Variables According to Physical Activity Frequency in Leisure Time in People with Low Pain

Dependence relationships were found between all variables related to depression and PAF in people with low pain, both in women (S-RD, *p* < 0.001; PHQ-8 DS, *p* < 0.001; depression symptoms, *p* < 0.001; and depression types, *p* < 0.001) and in men (S-RD, *p* = 0.002; PHQ-8 DS, *p* < 0.001; depression symptoms, *p* < 0.001; and depression types, *p* < 0.001). Table S2a,b show the associations between PAF and depression variables, as well as the prevalence of each of the conditions as a function of PAF and by sex, and the differences in proportions found. The highest S-RD, PHQ-8 DS, depression symptoms, and depression type scores were found in people who never performed PA.

**Figure 2:**
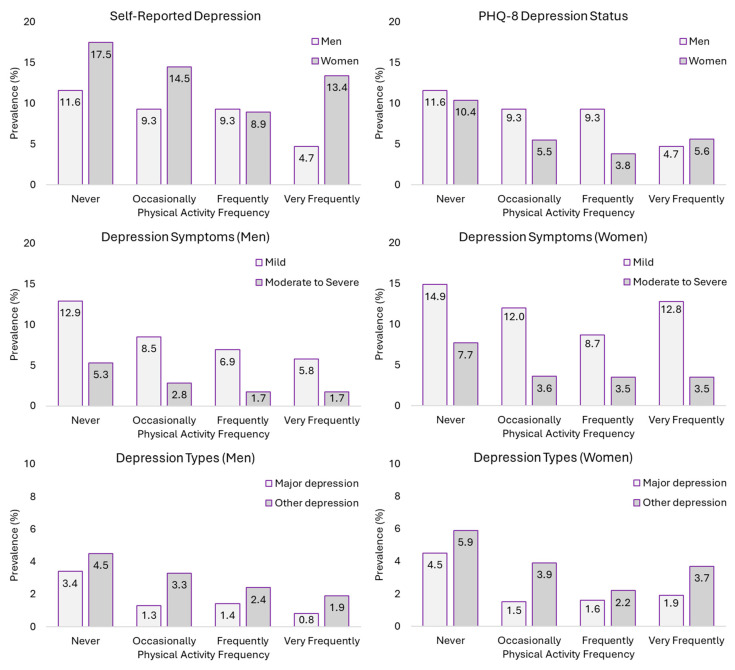
Prevalence of self-reported depression, PHQ-8 depression status, depression symptoms, and depression types according to physical activity frequency in people with low pain.

In men, the prevalence of self-reported depression was lower when PAF was higher, ranging from 11.6% in those who never performed PA to 4.3% in those who performed it very frequently. The same happened in PHQ-8 DS prevalence, ranging from 7.9% to 2.8% in those who performed PA never to very frequently. In contrast, women did not show the same trend in prevalence. While the highest prevalence was found in those who never performed PA (S-RD: 17.5%; PHQ-8 DS: 10.4%), the lowest prevalence was found in those who performed it frequently (S-RD: 8.9%; PHQ-8 DS: 3.8%), with an increase in prevalence in those who performed it very frequently (S-RD: 13.4%; PHQ-8 DS: 5.6%). These prevalence scores are shown in Figure 2.

Similar findings were found for depression symptoms and types. Men had a lower prevalence of all symptoms when PAF was higher. However, this was not the case for women. In women, the prevalence of mild symptoms of depression and other types of depression were highest in those who never performed PA (mild symptoms: 14.9%; other depression: 5.9%), and the lowest prevalence was seen in those who performed PA frequently (mild symptoms: 8.7%; other depression: 2.2%), again with an increase in those who performed PA very frequently (mild symptoms: 12.8%; other depression: 3.7%) (Figure 2).

### 3.3. Depression Variables According to Physical Activity Frequency in Leisure Time in People with Medium Pain

Table S3a,b show the associations found between PAF and depression-related variables in people with medium pain, in addition to the prevalence of S-RD, PHQ-8 DS, depression symptoms, and depression types according to PAF. Dependence relationships were found for all variables and sex (*p* < 0.001), except for PAF and self-reported depression in men (*p* = 0.108).

**Figure 3:**
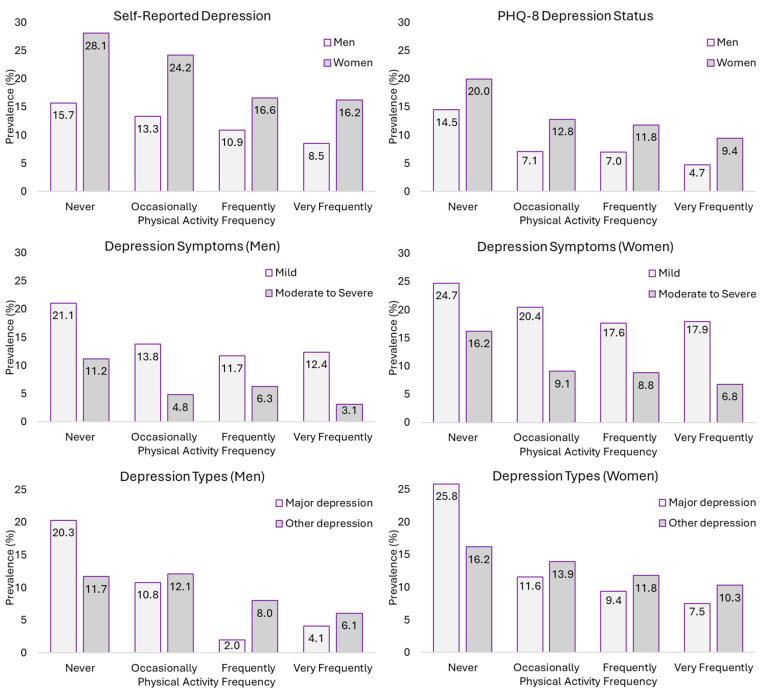
Prevalence of self-reported depression, PHQ-8 depression status, depression symptoms, and depression types according to physical activity frequency in people with medium pain.

In both men and women, the highest prevalence of S-RD and PHQ-8 DS was found in those who never performed PA, with the highest prevalence found in those who performed it very frequently. In men, S-RD prevalence in those who never performed PA was 15.7% vs. 8.5% in those who performed it very frequently. Something similar was found in PHQ-8 DS prevalence; men who never performed PA had a prevalence of 14.5% vs. 4.7% in those who performed it very frequently. In women, this prevalence was higher, although it was also found that the highest and lowest S-RD prevalence was found in those who never performed PA and those who performed PA very frequently (28.1% vs. 16.2%). The same happened in the prevalence of PHQ-8 DS, with those who never performed PA having the highest prevalence (20.0%) and those who performed it very frequently having the lowest (9.4%) (Figure 3).

As was the case for people with low pain, a higher prevalence of depressive symptoms and of the different types of depression was also found in people with medium pain who never performed PA, with lower prevalences found at higher PAF levels. The prevalence of mild symptoms was 21.1% and 24.7% in inactive men and women, compared to a prevalence of around 12% (11.7% and 12.4% in men) and 17% (17.6% and 17.9% in women) in men and women who performed PA frequently and very frequently. Likewise, the prevalence of major depression was also found to be higher in inactive men (20.3%) and women (25.8%) (Figure 3).

### 3.4. Depression Variables According to Physical Activity Frequency in Leisure Time in People with High Pain

**Figure 4:**
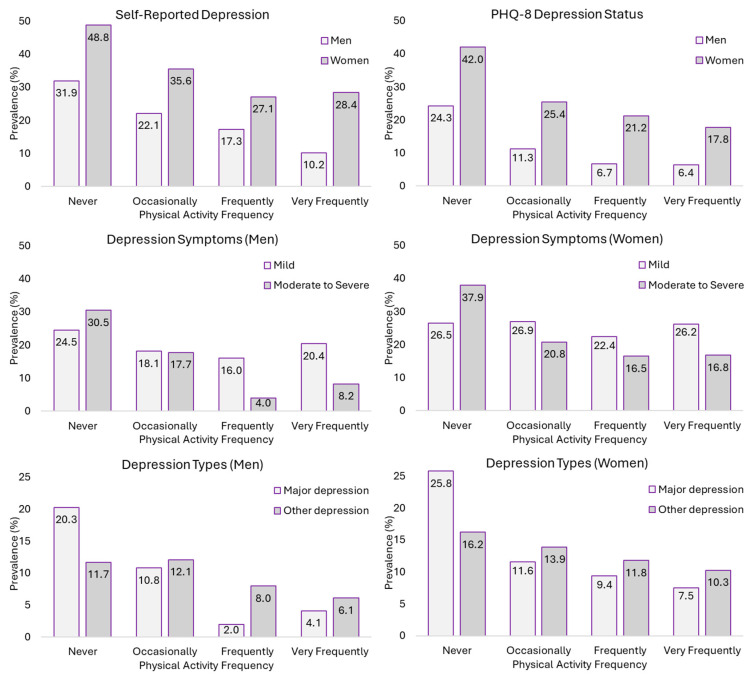
Prevalence of self-reported depression, PHQ-8 depression status, depression symptoms, and depression types according to physical activity frequency in people with high pain.

Finally, in people experiencing high levels of pain, dependence relationships were found between all depression-related variables and PAF (*p* < 0.001) (Table S4a,b). S-RD prevalence was as high as 48.8% and 31.9% in women and men who never performed PA. In women, the lowest prevalence was found in the group that performed PA frequently (27.1%), whereas in men, it was found in the group that performed it very frequently (10.2%). As for PHQ-8 DS, its prevalence was lower when PAF was higher, ranging from 42% and 24.3% in physically inactive women and men to 17.8% and 6.4% in the very active groups (Figure 4).

### 3.5. Regression Analysis for Self-Reported Depression and PHQ-8 Depression Status

**Figure 5:**
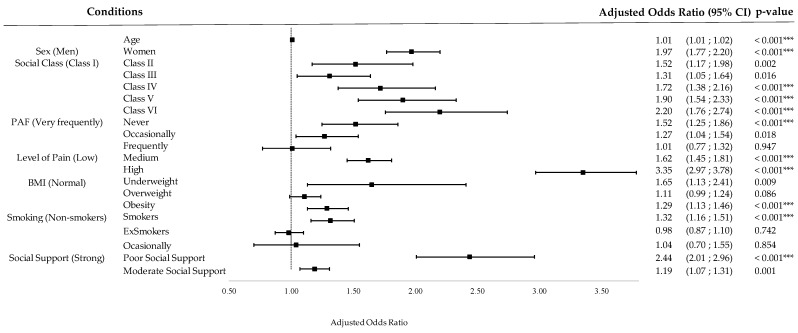
Regression analysis for S-RD: forest plot. CI (Confidence Interval); *** (*p*-value < 0.001).

The full results of the binary multiple logistic regression for S-RD are shown in Table S5. The model explains 13.7% of the variance (R^2^). Figure 5 shows the adjusted Odds Ratios (ORs) and their confidence intervals for each of the variables and their conditions. PAF in leisure time was found to be significantly associated with S-RD: compared to those who performed PA very frequently, those who never performed PA had an OR of 1.52 (95% CI: 1.25–1.82, *p* < 0.001), while those who performed PA occasionally had an OR of 1.27 (1.04–1.54, *p* < 0.001).

**Figure 6:**
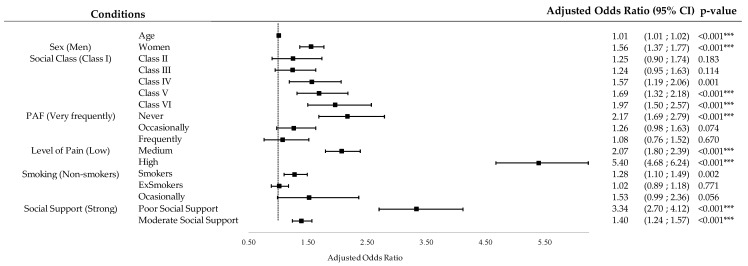
Regression analysis for PHQ-8 DS: forest plot. CI (Confidence Interval); *** (*p*-value < 0.001).

Table S6 shows the results of the multiple binary logistic regression for PHQ-8 DS. The model explained 17.5% of the variance (R^2^). PAF was significantly related to PHQ-8 DS: those who were inactive had a higher risk of being depressed (OR: 2.17, 95% CI: 1.69–2.79, *p* < 0.001). The adjusted ORs and their 95% CIs are shown in Figure 6.

## 4. Discussion

This study aimed to assess the dependence relationships between self-reported depression, depression status, depressive symptoms, depression types, and PAF in leisure time, as well as to compare these depression-related variables as a function of PAF in middle-aged and older Spanish adults with different pain levels (low, medium, and high). Risk factors for reporting depression and depression status were also assessed as secondary objectives.

### 4.1. Summary of Main Findings

#### 4.1.1. Sex and Depression

Regarding the impact of sex on depression, our results show that women had higher proportions of S-RD (24.0% vs. 13.0%), PHQ-8 DS (15.6% vs. 9.5%), mild (18. 8% vs. 13.2%) and moderate to severe depressive symptoms (12.6% vs. 7.2%), and major (7.6% vs. 4.4%) and other types of depression (8.1% vs. 5.2%). These findings are in line with other studies in the available scientific literature. Women are found to have a higher prevalence of depression than men [23]. Particularly, it is suggested that women are up to twice as likely to self-report depressive symptoms than men [24]. Shi et al. [23] state that women are more likely to report mild to moderate depressive symptoms, which is consistent with the results of the present study. Consistent with our results in the chronic pain population, a higher depression prevalence has also been found in women [19,25]. These differences could be related to the presence of biopsychosocial factors such as catastrophising, coping strategies, and hormonal and cultural factors [23].

#### 4.1.2. Physical Activity in Leisure Time and Depression

It should be noted that the most relevant findings were observed when analysing the prevalence of depression-related variables stratified by PAF. Dependence relationships were found between PAF and self-reported depression, depression status, depression symptoms, and depression types. These dependence relationships were found in all three pain level groups. For example, those who were never physically active (inactive) had the highest rates of self-reported depression and depression status in all three pain grades, low (between 7.9% and 17.5%, respectively), medium (between 14.5% and 28.1%) and high (between 31.8% and 48.8%). Although this finding was found in both physically active men and women, women had a higher prevalence of all variables related to depression. These results are supported in part by Christofaro et al.’s study [26] which reported that physically inactive people were more likely to show an association of depressive symptoms with pain in several body regions. Our results are also supported by Chen et al., who found a higher prevalence and higher risk of depression or a history of depression in people with chronic widespread pain and chronic regional pain who reported a low PAF [19]. People with chronic widespread pain and low PA were more likely to experience depression; specifically, people with low PA (in comparison to high PA) had a 1.73 (1.29 to 2.32) times higher likelihood of depression and were 1.43 (1.18 to 1.72) times more likely to have a history of depression [19]. In addition to these findings, previous studies have also shown a higher proportion of depressive symptoms in various physically inactive populations [27,28].

Considering the depression types reported by people with pain, major depression was found in higher and lower proportions in inactive people and in those who performed PA very frequently, respectively, in the group with low pain (men, 3.4% to 0.8%; women, 4.5% to 1.9%), in the group with medium pain (men, 6.1% to 2.3%; women, 9.7% to 2.1%), and in the group with high pain (men, 20.3% to 4.1%; women, 28.5% to 7.5%). These results are in line with those of the De Görgülü et al. [29], who reported that moderate to vigorous PA was associated with lower depressive symptoms in people with major depression. Previous studies also show that people with major depression are less physically active [28].

#### 4.1.3. Relationship between Depression and Sociodemographic, Clinical, and Lifestyle Variables

A multivariate logistic regression analysis was performed to examine the factors influencing the prevalence of depression in our sample of the Spanish population with pain. After adjusting the analysis for factors other than PAF, such as sex, age, social support, smoking, social class, BMI, and pain level, being physically inactive emerged as a significant risk factor for self-reported depression and depression status. Specifically, people who reported lower PAF (inactive compared to very frequently active) were more likely to have self-reported depression (OR: 1.52. 95% CI: 1.25–1.82) and depression status (OR: 2.17. 95% CI: 1.69–2.79).

On the other hand, the results show that there is a tendency for depression prevalence and depressive symptoms to be higher in men and women as the pain level increases. Our multivariate logistic regression analysis corroborated that pain level, adjusted for the other co-variables, was a significant risk factor for reporting depression. Specifically, people who reported a higher level or intensity of pain (high compared to mild) were 3.35 (95% CI: 2.97–3.78) times more likely to report depression and 5.4 (95% CI: 4.68–6.24) times more likely to report depression status. These results are supported by a previous two-year longitudinal follow-up study which observed that greater pain intensity at baseline was associated with an increased risk of developing depressive symptoms in adults [30]. This may suggest that PA may have a positive effect on reducing the symptoms and development of depression, which may be independent of the pain intensity of the individual [31].

Significant risk factors for an increased odds of depression were also found to be older age, being female, lower socio-economic class, lower or higher BMI, smoking, and low social support. Chen et al. found similar results, finding that women had a significantly higher risk of developing depression, influenced by factors such as age, socio-economic status, the presence of other comorbidities, and lifestyle factors [19].

### 4.2. Practical Implications

It is currently considered that pain and depression are comorbid disorders and the presence of depression in people with pain, especially in those with chronic pain, is highly prevalent. In addition, pain and depression play a major role in the development and maintenance of other chronic symptoms. In the treatment of these two conditions, pharmacological options such as tricyclic antidepressants currently appear to be the most effective in reducing pain [32]. However, there are some difficulties in their use. Among the problems related to mental health treatment is the lack of adherence or resistance to certain pharmacological treatments, which increases the need, from a public and community health perspective, to implement non-pharmacological treatments that complement pharmacological treatment and that are accessible to all people regardless of their socio-economic status. This is especially relevant in the post-pandemic era, as the pandemic has had a strong impact on mental health [33].

Therefore, when addressing depressive problems in people with pain from an integrated perspective, PA should be considered, as it could positively influence both aspects [29,34]. Moreover, public health programmes should promote other factors, such as social interactions or health habits.

### 4.3. Limitations and Future Lines of Research

This study has certain limitations that must be considered. It is a cross-sectional study, so no causal relationships can be established between the variables studied and an inverse relationship cannot be ignored. In addition, other variables, such as the use of antidepressants or other medications for the treatment of pain conditions that may also affect mood symptoms, such as immunomodulators, were not collected. Another limitation is that PAF was self-reported. Previous studies indicate that people with depression and greater pain intensity tend to underestimate their activity level [35]. Therefore, we recommend that these surveys include devices that can objectively measure participants’ PA, as is being done in other countries or other surveys [36]. It was not possible to analyse the extent of the pain symptoms, whether they affected one body area or several, and whether they were generalised, as the survey did not provide this information. Concerning these limitations, future studies should analyse mental health in people with different types of pain and make comparisons with the general population. Possible interactions with gender should also be analysed to investigate the optimal dose of PA for this population. Furthermore, longitudinal studies could establish the real impact of PA on people with pain and help to deepen knowledge in this field.

## 5. Conclusions

Associations were found between variables related to depression and physical activity frequency performed in leisure time in middle-aged and older Spanish populations with different pain levels. Inactive people with low, medium, and severe pain levels have higher proportions of self-reported depression, higher depression status prevalence, and higher rates of depressive symptoms and types than people who perform physical activity frequently or very frequently.

Together with a higher pain level, being female, from a lower social class, having poor perceived social support, smoking, and other sociodemographic variables, physical inactivity is presented as a risk factor for self-reported depression and depression status in middle-aged and older Spanish populations with pain.

It would be advisable to implement comprehensive prevention and intervention programmes for depression and depressive symptoms in middle-aged and older people, including frequent and very frequent physical activity in their leisure time and providing them with social and other support. Further research is needed to find the optimal type of programme and dosage for each person, considering their pain type and degree, as well as sex-related factors, among others.

## Figures and Tables

**Table 1:** Descriptive analysis.

		Overall = 13,686		Men = 5274		Women = 8412					
Variables		Median	IQR	Median	IQR	Median	IQR	X^2^	df	pU	V or φ
Age		60	19	59	19	60	20	-	-	<0.001	-
OSSS-3 sum		12	2	12	2	12	2	-	-	0.001	-
OSSS-3 level	Poor	624	4.70%	252	4.90%	372	4.50%	6.5	2	0.039	0.022
	Moderate	4630	34.70%	1834	35.80%	2796 *	34.00%				
	Strong	8074	60.60%	3030	59.20%	5044 *	61.40%				
Pain level	Low	6707	49.00%	2959	56.10%	3748 ***	44.60%	199.3	2	<0.001	0.121
	Moderate	4499	32.90%	1592	30.20%	2907 ***	34.60%				
	High	2480	18.10%	723	13.70%	1757 ***	20.90%				
Self-reported depression	Yes	2707	19.80%	687	13.00%	2020 ***	24.00%	246.8	1	<0.001	0.134
	No	10965	80.20%	4582	87.00%	6383 ***	74.60%				
PHQ-8 depression status	Yes	1806	13.30%	500	9.50%	1306 ***	15.60%	103.8	1	<0.001	0.087
	No	11782	86.70%	4738	90.50%	7044 ***	84.40%				
PHQ-8 depression symptoms	None	9895	72.80%	4167	79.60%	5278 ***	68.60%	201.7	2	<0.001	0.122
	Mild	2259	16.60%	692	13.20%	1567 ***	18.80%				
	Moderate to Severe	1434	10.60%	379	7.20%	1055 ***	12.60%				
PHQ-8 depression types	Major	861	6.30%	230	4.40%	631 ***	7.60%	104.4	2	<0.001	0.088
	Other Depression	945	7.00%	270	5.20%	675 ***	8.10%				
	None	11782	86.70%	4738	90.50%	7044 ***	84.40%				
PAF	Never	5566	40.70%	1997	37.90%	3569 ***	42.40%	40.5	3	<0.001	0.054
	Occasionally	5783	42.30%	2264	42.90%	3519	41.80%				
	Frequently	1075	7.90%	471	8.90%	604 ***	7.20%				
	Very Frequently	1262	9.20%	542	10.30%	720 **	8.60%				
BMI_Group	Underweight	167	1.30%	33	0.60%	134 ***	1.70%	308.9	3	<0.001	0.154
	Normal	4584	35.10%	1393	27.10%	3191 ***	40.30%				
	Overweight	5302	40.60%	2484	48.40%	2818 ***	35.60%				
	Obesity	2992	22.90%	1222	23.80%	1770	22.40%				
Smoking Status	Smokers	2818	20.60%	1335	25.30%	1483 ***	17.60%	1172.9	3	<0.001	0.293
	Ex Smokers	4082	29.80%	2251	42.70%	1831 ***	21.80%				
	Occasional Smokers	231	1.70%	118	2.20%	113 ***	1.30%				
	Non-Smokers	6550	47.90%	1568	29.70%	4982 ***	59.20%				
Social_Class	I	1275	9.60%	526	10.10%	749	9.30%	94.6	5	<0.001	0.084
	II	969	7.30%	350	6.70%	619 *	7.70%				
	III	2542	19.20%	926	17.70%	1616 **	20.10%				
	IV	1995	15.00%	907	17.40%	1088 **	13.50%				
	V	4478	33.70%	1854	35.50%	2624 ***	32.60%				
	VI	2015	15.20%	654	12.50%	1361 ***	16.90%				

n—participants; %—percentage; X^2^—Pearson chi-squared; df—degree of freedom; pU—*p*-value from Mann–Whitney U test; pX—*p*-value from chi-squared test; φ—phi coefficient; V—Cramer’s V coefficient; *—significant differences between sex proportions with *p* < 0.05 from pairwise z-test for independent proportions; **—*p* < 0.01; ***—*p* < 0.001; OSSS-3—Oslo Social Support Scale-3; Sum—summation.

## Data Availability

The original contributions presented in this study are included in the article/Supplementary Materials; further inquiries can be directed to the corresponding authors.

## Supplementary Materials

The following supporting information can be downloaded at: https://www.mdpi.com/article/10.3390/jpm14080855/s1, Table S1. Description of social classes based on occupational occupation; Table S2. (a) Prevalence of self-reported depression and PHQ-8 depression status according to physical activity frequency in men and women; (b) prevalence of depression symptoms and depression types according to physical activity frequency in men and women; Table S3. (a) Prevalence of self-reported depression and PHQ-8 depression status according to physical activity frequency in men and women; (b) prevalence of depression symptoms and depression types according to physical activity frequency in men and women; Table S4. (a) Prevalence of self-reported depression and PHQ-8 depression status according to physical activity frequency in men and women; (b) prevalence of depression symptoms and depression types according to physical activity frequency in men and women. Table S5. Regression analysis: self-reported depression. Table S6. Regression analysis: PHQ-8 depression status.
