# Supplementary material for: Relationship between Depression and Physical Activity Frequency in Spanish People with Low, Medium, and High Pain Levels
Source: J Pers Med. 2024 Aug 12;14(8):855. doi: 10.3390/jpm14080855 (PMC11355638; doi:10.3390/jpm14080855)
Supplement: Supplementary file 1 [file jpm-14-00855-s001.zip › Table S1. Description of social classes based on occupational occupation.pdf]

Table S1. Description of social classes based on occupational occupation.

**CLASS I** - Directors and managers of establishments with 10 or more employees, and professionals traditionally associated with university degrees
**CLASS II** - Directors and managers of establishments with fewer than 10 employees, professionals traditionally associated with university degrees and other technical support professionals. technical support professionals. Athletes and artists
**CLASS III** - Intermediate occupations and self-employed workers
**CLASS IV** - Supervisors and workers in skilled technical occupations
**CLASS V** - Skilled workers in the primary sector and other semi-skilled workers semi-skilled workers
**CLASS VI** - Unskilled workers
