# Supplementary material for: Relationship between Depression and Physical Activity Frequency in Spanish People with Low, Medium, and High Pain Levels
Source: J Pers Med. 2024 Aug 12;14(8):855. doi: 10.3390/jpm14080855 (PMC11355638; doi:10.3390/jpm14080855)
Supplement: Supplementary file 1 [file jpm-14-00855-s001.zip › Table S2a and S2b. Relationship between Depression variables and Physical Actity Frequency in people with Low Pain.pdf]

Table S2.a Prevalence of Self-Reported Depression and PHQ-8 Depression Status according to Physical Activity Frequency in men's and women's.

**Variables**	PAF																							
Self-reported Depression	**Never (A)**				**Occasionally (B)**				**Frequently (C)**				**Very Frequently (D)**				**X²**		**df**		**p**		**V**	
	Women		Men		Women		Men		Women		Men		Women		Men		Women	Men	Women	Men	Women	Men	Women	Men
	n	%	n	%	n	%	n	%	n	%	n	%	n	%	n	%								
No	1067	82.5	854	88.4	1508	85.5	1215	90.7	286	91.1	263	90.7	324	86.6	334	95.3	16.8	14.7	3	3	<.001	0.002	0.070	0.070
Yes	227	17.5	112	11.6	256	14.5	124	9.3	28	8.9	27	9.3	50	13.4	17	4.3								
Proportions' differences post hoc (between frequency of physical activity)																								
Proportions's differences p-value									A (.001)<br>B (.047)															
	C (.001)		D (.001)		C (.047)		D (.032)																	
**Variables**	PAF																							
PHQ-8 Depression Status	**Never (A)**				**Occasionally (B)**				**Frequently (C)**				**Very Frequently (D)**				**X²**		**df**		**p**		**V**	
	Women		Men		Women		Men		Women		Men		Women		Men		Women	Men	Women	Men	Women	Men	Women	Men
	n	%	n	%	n	%	n	%	n	%	n	%	n	%	n	%								
No	1157	89.6	884	92.1	1660	94.5	1273	95.4	300	96.2	277	96.2	353	94.4	352	97.2	34.6	20.1	3	3	<.001	<.001	0.096	0.083
Yes	134	10.4	76	7.9	96	5.5	61	4.6	12	3.8	11	3.8	21	5.6	10	2.8								
Proportions' differences post hoc (between frequency of physical activity)																								
Proportions's differences p-value					A (<.001)		A (.005)		A (.002)				A (.031)		A (.004)									
	B (<.001)<br>C (.002)<br>D (.031)		B (.005)<br>D (.004)		C (.047)		D (.032)																	

p (p-value from pairwise z-test for independent proportions between frequency of physical activity in women and men); ** ($p<0.01$); *** ($p<0.001$); X^2 (Chi-Square); df (Degree freedom); V (V's Cramer coefficients).

Table S2.b Prevalence of Depression Symptoms and Depression Types according to Physical Activity Frequency in men’s and women’s.

**Variables**	PAF																							
Depression Symptoms	**Never (A)**				**Occasionally (B)**				**Frequently (C)**				**Very Frequently (D)**				**X^2**		**df**		**p**		**V**	
	Women		Men		Women		Men		Women		Men		Women		Men		Women	Men	Wome<br>n	Men	Women	Men	Women	Men
	n	%	n	%	n	%	n	%	n	%	n	%	n	%	n	%								
None	1000	77.5	785	81.8	1482	84.4	1184	88.8	274	87.8	263	91.3	313	83.7	33<br>5	92.5	43.2	43.6	6	6	<.001	<.001	0.108	0.122
Mild	192	14.9	124	12.9	210	12.0	113	8.5	27	8.7	20	6.9	48	12.8	21	5.8								
Highs	99	7.7	51	5.3	640	3.6	37	2.8	11	3.5	5	1.7	13	3.5	6	1.7								

Proportions’ differences post hoc (between frequency of physical activity)

	Never (A) Women	Never (A) Men	Occasionally (B) Women	Occasionally (B) Men	Frequently (C) Women	Frequently (C) Men	Very Frequently (D) Women	Very Frequently (D) Men
Proportions's differences p-values			A (<.001)	A (<.001)	A (.001)	A (.001)		A (.001)
	C (.025)	B (.003)<br>C (.032)<br>D (.001)						
	B (<.001)<br>D (.026)	B (.011)<br>D (.021)						

**Variables**	PAF																							
Depression Types	**Never (A)**				**Occasionally (B)**				**Frequently (C)**				**Very Frequently (D)**				**X^2**		**df**		**p**		**V**	
	Women		Men		Women		Men		Women		Men		Women		Men		Women	Men	Wome<br>n	Men	Women	Men	Women	Men
	n	%	n	%	n	%	n	%	n	%	n	%	n	%	n	%								
Major	58	4.5	33	3.4	27	1.5	17	1.3	5	1.6	4	1.6	7	1.9	3	0.8	40.7	24.4	6	6	<.001	<.001	0.074	0.064
Other	76	5.9	43	4.5	69	3.9	44	3.3	7	2.2	7	2.2	14	3.7	7	1.9								
None	1157	89.6	884	92.1	1660	94.5	1273	95.4	300	96.2	277	96.2	353	94.4	36<br>2	97.2								

Proportions’ differences post hoc (between frequency of physical activity)

	Never (A) Women	Never (A) Men	Occasionally (B) Women	Occasionally (B) Men	Frequently (C) Women	Frequently (C) Men	Very Frequently (D) Women	Very Frequently (D) Men
Proportions's differences p-values	B (<.001)	B (.003)						
			A (<.001)	A (.005)	A (.002)		A (.031)	A (.004)

p (p-value from pairwise z-test for independent proportions between frequency of physical activity in women and men); Highs: Moderate to severe symptoms; X^2 (Chi-Square); df (Degree freedom); V (V's Cramer coefficients).
