# Supplementary material for: Relationship between Depression and Physical Activity Frequency in Spanish People with Low, Medium, and High Pain Levels
Source: J Pers Med. 2024 Aug 12;14(8):855. doi: 10.3390/jpm14080855 (PMC11355638; doi:10.3390/jpm14080855)
Supplement: Supplementary file 1 [file jpm-14-00855-s001.zip › Table S3a and S3b. Relationship between Depression variables and Physical Actity Frequency in people with Medium Pain.pdf]

Table S3.a Prevalence of Self-Reported Depression and PHQ-8 Depression Status according to Physical Activity Frequency in men's and women's.

**Variables**	PAF																							
Self-reported Depression	**Never (A)**				**Occasionally (B)**				**Frequently (C)**				**Very Frequently (D)**				**X^2**		**df**		**p**		**V**	
	Women		Men		Women		Men		Women		Men		Women		Men		Women	Men	Women	Men	Women	Men	Women	Men
	n	%	n	%	n	%	n	%	n	%	n	%	n	%	n	%								
No	919	71.9	542	84.3	897	75.8	598	86.7	171	83.4	115	89.1	197	83.8	118	91.5	24.7	6.1	3	3	<.001	.108	.092	.062
Yes	360	28.1	101	15.7	286	24.2	92	13.3	34	16.6	14	10.9	38	16.2	11	8.5								
Proportions' differences post hoc (between frequency of physical activity)																								
Proportions's differences p-value										A (.003)					A (.001)<br>B (.046)									
		C (.003)<br>D (.001)				C (.046)																		

**Variables**	PAF																							
PHQ-8 Depression Status	**Never (A)**				**Occasionally (B)**				**Frequently (C)**				**Very Frequently (D)**				**X^2**		**df**		**p**		**V**	
	Women		Men		Women		Men		Women		Men		Women		Men		Women	Men	Women	Men	Women	Men	Women	Men
	n	%	n	%	n	%	n	%	n	%	n	%	n	%	n	%								
No	1005	80	543	85.5	1032	87.2	638	92.9	180	88.2	19	93	212	90.6	123	95.3	34.8	26.1	3	3	.001	<.001	.110	.129
Yes	252	20	92	14.5	152	12.8	49	7.1	24	11.8	9	7	22	9.4	6	4.7								
Proportions' differences post hoc (between frequency of physical activity)																								
Proportions's differences p-value						A (<.001)		A (.0001)		A (.030)					A (.001)		A (.014)							
		B (<.001)<br>C (.030)<br>D (.001)		B (<.001)<br>D (.014)																				

p (p-value from pairwise z-test for independent proportions between frequency of physical activity in women and men); ** (p<0.01); *** (p<0.001); X^2 (Chi-Square); df (Degree freedom); V (V's Cramer coefficients).

Table S3.b Prevalence of Depression Symptoms and Depression Types according to Physical Activity Frequency in men's and women's.

**Variables**	PAF																							
Depression Symptons	**Never (A)**				**Occasionally (B)**				**Frequently (C)**				**Very Frequently (D)**				**X^2**		**df**		**p**		**V**	
	Women		Men		Women		Men		Women		Men		Women		Men		Women	Men	Women	Men	Women	Men	Women	Men
	n	%	n	%	n	%	n	%	n	%	n	%	n	%	n	%								
None	742	59	430	67.7	834	70.4	559	81.4	150	73.5	105	82	176	75.2	109	84.5	60.4	46.5	6	6	<.001	<.001	.102	.121
Milds	311	24.7	134	21.1	242	20.4	95	13.8	36	17.6	15	11.7	42	17.9	16	12.4								
Highs	204	16.2	71	11.2	108	9.1	33	4.8	18	8.8	8	6.3	16	6.8	4	3.1								
Proportions' differences post hoc (between frequency of physical activity)																								
Proportions's differences p-values						A (<.001)		A (<.001)		A (<.001)		A (.007)		A (<.001)		A (.001)								
				B (.003)																				
		B (<.001) C (.038) D (.001)		B (.001) D (.030)																				

**Variables**	PAF																							
Depression Types	**Never (A)**				**Occasionally (B)**				**Frequently (C)**				**Very Frequently (D)**				**X^2**		**df**		**p**		**V**	
	Women		Men		Women		Men		Women		Men		Women		Men		Women	Men	Women	Men	Women	Men	Women	Men
	n	%	n	%	n	%	n	%	n	%	n	%	n	%	n	%								
Major	122	9.7	39	6.1	63	5.3	20	2.9	9	4.4	5	3.9	5	2.1	3	2.3	40.3	26.7	6	6	<.001	<.001	0.084	0.092
Other	130	10.3	53	8.3	89	7.5	29	4.2	15	7.4	4	3.1	17	7.3	3	2.3								
None	1005	80	543	85.5	1032	87.2	638	92.9	180	88.2	119	93	212	90.6	123	95.3								
Proportions' differences post hoc (between frequency of physical activity)																								
Proportions's differences p-values		B (<.001) D (.001)		B (.027)																				
				B (.011)				A (<.001)								A (.014)								
						A (<.001)				A (.030)				A (.001)										

p (p-value from pairwise z-test for independent proportions between frequency of physical activity in women and men); Highs: Moderate to severe symptoms; X^2 (Chi-Square); df (Degree freedom); V (V's Cramer coefficients).
