# Supplementary material for: Relationship between Depression and Physical Activity Frequency in Spanish People with Low, Medium, and High Pain Levels
Source: J Pers Med. 2024 Aug 12;14(8):855. doi: 10.3390/jpm14080855 (PMC11355638; doi:10.3390/jpm14080855)
Supplement: Supplementary file 1 [file jpm-14-00855-s001.zip › Table S4a and S4b. Relationship between Depression variables and Physical Actity Frequency in people with High Pain.pdf]

Table S4.a Prevalence of Self-Reported Depression and PHQ-8 Depression Status according to Physical Activity Frequency in men’s and women’s.

**Variables**	PAF																							
Self-reported Depression	**Never (A)**				**Occasionally (B)**				**Frequently (C)**				**Very Frequently (D)**				**X²**		**df**		**p**		**V**	
	Women		Men		Women		Men		Women		Men		Women		Men		Women	Men	Women	Men	Women	Men	Women	Men
	n	%	n	%	n	%	n	%	n	%	n	%	n	%	n	%								
No	509	51.2	263	68.1	365	64.4	183	77.9	62	72.9	43	82.7	78	71.6	44	89.8	44.2	17	3	3	<.001	<.001	0.159	0.154
Yes	485	48.8	123	31.9	202	35.6	52	22.1	23	27.1	9	17.3	31	28.4	5	10.2								
Proportions's differences p-value						A (<.001)				A (.001)				A (<.001)		A (.010)								
		B (<.001)<br>C (.001)<br>D (<.001)		D (.010)																				
**Variables**	PAF																							
PHQ-8 Depression Status	**Never (A)**				**Occasionally (B)**				**Frequently (C)**				**Very Frequently (D)**				**X²**		**df**		**p**		**V**	
	Women		Men		Women		Men		Women		Men		Women		Men		Women	Men	Women	Men	Women	Men	Women	Men
	n	%	n	%	n	%	n	%	n	%	n	%	n	%	n	%								
No	571	58	261	68	419	74.6	179	77.2	67	78.8	45	90	88	82.2	44	89.8	64.9	21.5	3	3	<.001	<.001	0.193	0.173
Yes	143	42	123	32	143	25.4	53	22.8	18	21.2	5	10	19	17.8	5	10.2								
Proportions’ differences post hoc (between frequency of physical activity)																								
Proportions's differences p-value						A (<.001)				A (.001)		A (.008)		A (<.001)		A (.010)								
		B (<.001)<br>C (.001)<br>D (<.001)		C (.008)<br>D (.010)																				

p (p-value from pairwise z-test for independent proportions between frequency of physical activity in women and men); X² (Chi-Square); df (Degree freedom); V (V's Cramer coefficients).

Table S4.b Prevalence of Depression Symptoms and Depression Types according to Physical Activity Frequency in men's and women's.

Variables	PAF																							
Depression Symptons	Never (A)				Occasionally (B)				Frequently (C)				Very Frequently (D)				X^2		df		p		V	
	Women		Men		Women		Men		Women		Men		Women		Men		Women	Men	Women	Men	Women	Men	Women	Men
	n	%	n	%	n	%	n	%	n	%	n	%	n	%	n	%								
None	350	35.6	173	45.1	294	52.3	146	64.2	52	61.2	40	80	6	57	35	71.4	83	49.7	6	6	<.001	<.001	0.155	0.181
Milds	261	26.5	94	24.5	151	26.9	42	18.1	19	22.4	8	16	26.2	18	10	20.4								
Highs	373	37.9	117	30.5	117	20.8	41	17.7	14	16.5	2	4	18	16.8	4	8.2								
Proportions' differences post hoc (between frequency of physical activity)																								
Proportions's differences p-values						A (<.001)		A (<.001)		A (<.001)		A (.003)		A (<.001)		A (.003)								
		B (<.001)<br>C (<.001)<br>D (<.001)		B (.003)<br>C (<.001)<br>D (.006)																				
Variables	PAF																							
Depression Types	Never (A)				Occasionally (B)				Frequently (C)				Very Frequently (D)				X^2		df		p		V	
	Women		Men		Women		Men		Women		Men		Women		Men		Women	Men	Women	Men	Women	Men	Women	Men
	n	%	n	%	n	%	n	%	n	%	n	%	n	%	n	%								
Major	254	25.8	78	20.3	65	11.6	25	1.8	8	9.4	1	2	8	7.5	2	4.1	76.8	27.1	6	6	<.001	<.001	0.149	0.138
Other	159	16.2	45	11.7	78	13.9	28	12.1	10	11.8	4	8	11	10.3	3	6.1								
None	571	58	261	68	419	74.6	179	77.2	67	78.8	45	90	88	82.2	44	89.8								
Proportions' differences post hoc (between frequency of physical activity)																								
Proportions's differences p-values		B (<.001)<br>C (.004)<br>D (<.001)		B (.013)<br>C (.010)<br>D (.035)																				
						A (<.001)				A (.001)		A (.008)		A (<.001)		A (.010)								

p (p-value from pairwise z-test for independent proportions between frequency of physical activity in women and men); Highs: Moderate to severe symptoms; ** (p<0.01); *** (p<0.001); X^2 (Chi-Square); df (Degree freedom); V (V's Cramer coefficients).
