# Supplementary material for: Relationship between Depression and Physical Activity Frequency in Spanish People with Low, Medium, and High Pain Levels
Source: J Pers Med. 2024 Aug 12;14(8):855. doi: 10.3390/jpm14080855 (PMC11355638; doi:10.3390/jpm14080855)
Supplement: Supplementary file 1 [file jpm-14-00855-s001.zip › Table S5. Regression Self-Reported Depression.pdf]

Table S5. Multivariate binary logistic regression analysis including Self-Related Depression as the dependant variable.

	β	OR	C.I. (95%)	p
Age	0.014	1.01	(1.01; 1.02)	<0.001***
Sex (Men)				
Women	0.679	1.97	(1.77; 2.20)	<0.001***
SocialClass (I)		Ref.		
II	0.418	1.52	(1.17; 1.98)	0.002**
III	0.272	1.31	(1.05; 1.64)	0.016*
IV	0.545	1.72	(1.38; 2.16)	<0.001***
V	0.639	1.90	(1.54; 2.33)	<0.001***
VI	0.787	2.20	(1.76; 2.74)	<0.001***
PAF (Very frequently)		Ref.		
Never	0.422	1.52	(1.25; 1.86)	<0.001***
Occasionally	0.236	1.27	(1.04; 1.54)	0.018*
Frequently	0.009	1.01	(0.77; 1.32)	0.947
Pain Level (Low)		Ref.		
Medium	0.480	1.62	(1.45; 1.81)	<0.001***
High	1.209	3.35	(2.97; 3.78)	<0.001***
BMI (Normal)		Ref.		
Underweight	0.500	1.65	(1.13; 2.41)	0.009**
Overweight	0.100	1.11	(0.99; 1.24)	0.086
Obesity	0.250	1.29	(1.13; 1.46)	<0.001***
Smoking Status (NoSmokers)		Ref.		
Smokers	0.280	1.32	(1.16; 1.51)	<0.001***
ExSmokers	-0.020	0.98	(0.87; 1.10)	0.742
Ocassionally	0.038	1.04	(0.70; 1.55)	0.854
Social Support (Strong)		Ref.		
Poor	0.891	2.44	(2.01; 2.96)	<0.001***
Moderate	0.169	1.19	(1.07; 1.31)	0.001**
Constant	-4.250	0.01		

β (Beta); OR (Odds ratio); Ref. (Reference); C.I. (Confidence interval); p (p-value); * (p-value<0.05); ** (p-value<0.01); *** (p-value<0.001).
