# Supplementary material for: Relationship between Depression and Physical Activity Frequency in Spanish People with Low, Medium, and High Pain Levels
Source: J Pers Med. 2024 Aug 12;14(8):855. doi: 10.3390/jpm14080855 (PMC11355638; doi:10.3390/jpm14080855)
Supplement: Supplementary file 1 [file jpm-14-00855-s001.zip › Table S6. Regression PHQ-8 Status Depression.pdf]

Table S6. Multivariate binary logistic regression analysis including PHQ-8 Depression Status as the dependant variable.

	β	OR	C.I. (95%)	p
Age	0.013	1.01	(1.01; 1.02)	<0.001***
Sex (Men)		Ref.		
Women	0.444	1.56	(1.37; 1.77)	<0.001***
SocialClass (I)		Ref.		
II	0.224	1.25	(0.90; 1.74)	0.183
III	0.219	1.24	(0.95; 1.63)	0.114
IV	0.448	1.57	(1.19; 2.06)	0.001**
V	0.527	1.69	(1.32; 2.18)	<0.001***
VI	0.686	1.97	(1.50; 2.57)	<0.001***
PAF (Very frequently)		Ref.		
Never	0.776	2.17	(1.69; 2.79)	<0.001***
Occasionally	0.234	1.26	(0.98; 1.63)	0.074
Frequently	0.075	1.08	(0.76; 1.52)	0.670
Pain Level (Low)		Ref.		
Medium	0.727	2.07	(1.80; 2.39)	<0.001***
High	1.687	5.40	(4.68; 6.24)	<0.001***
Smoking Status (NoSmokers)		Ref.		
Smokers	0.246	1.28	(1.10; 1.49)	0.002**
ExSmokers	0.021	1.02	(0.89; 1.18)	0.771
Ocassionally	0.424	1.53	(0.99; 2.36)	0.056
Social Support (Strong)		Ref.		
Poor	1.205	3.34	(2.70; 4.12)	<0.001***
Moderate	0.334	1.40	(1.24; 1.57)	0.001**
Constant	-4.872	0.01		

β (Beta); OR (Odds ratio); Ref. (Reference); C.I. (Confidence interval); p (p-value); * (p-value<0.05); ** (p-value<0.01); *** (p-value<0.001).
